# Ovarian cancer subtypes and survival in relation to three comprehensive imaging parameters

**DOI:** 10.1186/s13048-020-00625-8

**Published:** 2020-03-07

**Authors:** Hanna Sartor, Maria Bjurberg, Mihaela Asp, Anna Kahn, Jenny Brändstedt, Päivi Kannisto, Karin Jirström

**Affiliations:** 1 Diagnostic Radiology, Department of Translational Medicine, Lund University, Skåne University Hospital, Lund, Sweden; 2 Oncology and Pathology, Department of Clinical Sciences, Lund University, Lund, Sweden; 3 Obstetrics and Gynecology, Department of Clinical Sciences, Lund University, Lund, Sweden

**Keywords:** Ovarian Cancer, Peritoneal Carcinomatosis, Lymph nodes, Breast density, Survival analyses

## Abstract

**Background:**

Ovarian cancer (OC) is usually detected in late clinical stages, and imaging at diagnosis is crucial. Peritoneal carcinomatosis (PC) and cardio phrenic lymph nodes (CPLN) are pathological findings of computed tomography (CT) and are relevant for surgical planning. Furthermore, mammographic breast density (BD) has shown an association with OC risk and might be prognostically relevant. However, it is not known if PC, CPLN, and BD are associated with aggressive OC subtypes and impaired OC survival. Herein, we investigated associations between three comprehensive image parameters and OC subtypes and survival.

**Methods:**

The Malmö Diet and Cancer Study is a prospective study that included 17,035 women (1991–1996). Tumor information on 159 OC and information on OC specific survival (last follow-up, 2017-12-31) was registered. The CT and mammography closest to diagnosis were evaluated (Peritoneal Carcinomatosis Index PCI, CPLN, and BD). Associations between CT-PCI, CPLN, and BD vs. clinical stage [stage I vs. advanced stage (II-IV), histological type/grade (high grade serous and endometrioid vs. other subtypes], and OC-specific survival were analyzed by logistic and Cox regression.

**Results:**

There was a significant association between higher CT-PCI score and advanced clinical stage (adjusted OR 1.26 (1.07–1.49)), adjusted for age at diagnosis and histological type/grade. Increasing CT-PCI was significantly associated with impaired OC specific survival (adjusted HR 1.04 (1.01–1.07)), adjusted for age at diagnosis, histological type/grade, and clinical stage. There was no significant association between PCI and histological type/grade, nor between BD or CPLN vs. the studied outcomes.

**Conclusions:**

Image PCI score was significantly associated with advanced clinical stages and impaired OC survival. An objective approach (based on imaging) to scoring peritoneal carcinomatosis in ovarian cancer could help surgeons and oncologists to optimize surgical planning, treatment, and care.

## Background

Ovarian cancer is the seventh most common cancer and the eighth leading cause of death from cancer in women globally, and its incidence rates are highest in more developed regions [1]. Also, women are often diagnosed at a late stage of the disease since ovarian cancer presents late and with diffuse clinical symptoms, such as vague abdominal pain or malaise [2]. Due to the late diagnosis, it is crucial to optimize imaging and treatment at time of diagnosis.

Computed tomography (CT) is often the first test with which ovarian cancer is detected [3]. One of the most common findings is peritoneal metastases that can be evaluated with CT with an overall sensitivity of more than 90% (depending on anatomical site, lesion size, and radiologist experience) and with high specificity (around 80%) as compared to findings at surgery [4, 5]. The Sugarbaker peritoneal carcinomatosis index (PCI) [6] allows a quantification of peritoneal metastases both surgically [7] and with imaging [5], and previous studies have reported that CT-PCI has the potential to help evaluate surgical outcome [8] and the 5-year survival probability in women with advanced ovarian cancer [9].

Furthermore, cardio phrenic lymph nodes (CPLN) are located above the abdominal cavity and diaphragm and are, by convention, considered radiologically positive if the short axis in the transaxial plane is ≥5 mm. However, how they should be handled in surgical care and their role in ovarian cancer prognosis have been debated [10, 11]. Interestingly, a recent paper (predominantly high grade serous OC) [10] indicated that there was a stronger correlation between CPLN positivity and peritoneal carcinomatosis of the upper abdomen (especially of the diaphragm) than between CPLN positivity and abdominal lymph node status, which makes the two parameters (CT-PCI and CPLN) particularly interesting to study together.

Women in developed countries are frequently subject to imaging as part of breast cancer-screening programs that include mammography. Mammographic breast density (BD) is an image parameter that reflects the composition of the breast tissue, and women with dense breasts have a higher incidence of breast cancer than do women with non-dense breasts and possibly also a worse prognosis [12, 13]. Also, increased BD has also been shown to be associated with a modestly increased risk of ovarian cancer [14]. However, it is not known if BD is linked to more aggressive types of ovarian cancer in terms of stage, histological grade, and survival, and this has not been previously studied.

Imaging is an underused biomarker since it has the potential to aid in the prediction of clinical decision making and prognosis. However, currently, in clinical routines addressing ovarian cancer, imagining is not used in a structured manner. However, it is a challenge to select relevant imaging parameters, which must be both readily available and pragmatic to analyze for the radiologist. The joint initiative on several imaging parameters in relation to long term follow up in ovarian cancer has the possibility to represent such relevant imaging parameters and has, to the best of our knowledge, not previously been investigated.

The goal of this study was to analyze three comprehensive imaging parameters available for most women with ovarian cancer, with the hypothesis that these might add information on ovarian cancer subtypes and survival at an early diagnostic stage.

## Methods

### The Malmö diet and Cancer study (MDCS)

The MDCS (LU 51–90) and the present study (Dnr 530/2008) were approved by the regional ethics committee in Lund, Sweden. All women gave written informed consent.

The MDCS [15–17] was a population-based, prospective cohort study, which included 17,035 women during 1991–1996. Various baseline data were registered (e.g., parity, oral contraceptives), and ovarian cancer cases were identified prospectively from the MDCS cohort. The associated pathological (histological subtype and grade, clinical stage) and radiological variables at ovarian cancer diagnosis (mammographic density, CT-PCI, CPLN) were collected and added to the database retrospectively for research purposes. Cause of death (ovarian cancer as an underlying or subordinate cause of death) and vital status (alive or dead from another cause was classified as alive) was registered with the last follow-up on 31 December 2017 (mean follow up time, 16.7 years).

### CT-PCI and CPLN

Patients with digital CT images were eligible for image analyses. Hence, patients with analogue CT images are classified as “missing CT” (Table 1). All patients underwent CT in the supine position and the majority with intravenous and oral contrast. Images were reformatted in the coronal and sagittal planes. The images were gathered over a long period of time. Hence, there is a variety of radiology systems; this has, however, been shown not to affect peritoneal carcinomatosis detection [4]. The median time between CT closest to diagnosis and registered OC diagnosis was 6 days (range 152 days). CT-PCI was retrospectively scored using the Sugarbaker classification [5, 6] by one specialist in radiology with subspeciality training in gastroradiology (CB). The PCI is calculated as the sum of numerical lesion scores assigned to 13 abdominopelvic regions. The lesion score relates to the largest visible implant. CPLN was defined as pathologically enlarged if measuring ≥5 mm in the short axis in the axial plane [10] and was defined as negative or positive (i.e., enlarged). The evaluation of CPLN was made on the same CT as for CT-PCI and was retrospectively analyzed by one radiologist with subspecialty training in gastroradiology (AK).

**Table 1:** Imaging parameters and patient and tumor characteristics in relation to ovarian cancer-specific survival

	Alive	Dead
Age at diagnosis		
n	58	101
median (range)	68 (37)	68 (40)
Missing	0	0
Parity		
Nullipara	8 (14.0)	23 (23.2)
Yes	49 (86.0)	76 (76.8)
Missing	1	2
Oral contraceptives		
No	31 (53.5)	54 (53.5)
Yes	27 (46.5)	47 (46.5)
Missing	0	0
Density		
Low	22 (50)	29 (46.8)
High	22 (50)	33 (53.2)
Missing	14	39
Stage		
Stage I	19 (32.8)	7 (7.2)
Advanced Stage	39 (67.2)	90 (92.8)
Missing	0	4
Histological type/grade		
Low grade serous/other	22 (38.6)	24 (24.7)
High grade serous and all endometroid	35 (61.4)	73 (75.3)
Missing	1	4
PCI		
median (range)	2.5 (39)	17.5 (39)
Missing CT	16	49
CPLN		
< 5 mm	24 (57.1)	22 (42.3)
≥5 mm	18 (42.9)	30 (57.7)
Missing CT	16	49

Categorical variables are presented as count (percent) and continuous variables are presented as median (range)

### Breast density

BD was retrospectively graded in one of four categories (a-d) according to Breast Imaging Reporting and Data System 5th Ed (BI-RADS) [18] using the screening mammography (analogue (before year 2004) or digital) closest before ovarian cancer diagnosis (median, 1.2 years). The BD was estimated using both breasts and all views. All mammograms were assessed by one specialist in radiology with special training in BD (HS).

### Tumor characteristics and clinical stage

All tumors between 1991 and 2007 were reevaluated by a senior pathologist (KJ) regarding histological subtype and histological grade [19]. Information on tumors from 2008 and onward were extracted from the original pathology reports, with the reports being more structured from these time points and onwards. Of the 166 OC cases (100%), 7 (4%) cases were defined as non-epithelial and excluded. Of the 159 OC cases eligible for analysis, the distribution was as follows: 99 (60%) were classified as serous, 27 (16%) endometrioid, ten (6%) mucinous, seven (4%) clear-cell, and 12 (7%) undifferentiated/adenocarcinoma NOS, and four cases (2%) had missing information on histologic subtype in medical charts or pathology reports. No borderline tumors were included in the study, as none had been registered. Tumor grade was divided into low or high, with the previous intermediate grade classified as high grade in this present study. Because of the small number of cases and for purposes of clinical relevance, histological subtype and grade were combined into one variable with the following classification: high-grade serous tumors and endometroid tumors (all grades) were grouped, and all other histological types combined in one group (including serous type with low or unknown grade). Information regarding the clinical stage was obtained retrospectively from the medical charts, following the standardized WHO classification of tumor staging and classified as stage I or advanced stage (II-IV).

### Statistics

Logistic regression was used to analyze CT-PCI (continuous), dichotomized BD [fatty (a + b) vs. dense (c + d)], and CPLN (binary) in relation to histologic type/grade (binary) and clinical stage (binary), which yielded odds ratios (OR) and 95% confidence intervals (CI). Adjustments were made for age at diagnosis (continuous), oral contraceptive use at baseline (binary), and parity (binary) in density analyses. Adjustments (CT-PCI and CPLN) were made for age at diagnosis (continuous) and histologic subtype/grade or clinical stage (when the variable was not an endpoint). Kaplan-Meier estimates were used to present survival probabilities for breast density and CPLN, respectively. Associations between selected imaging parameters and ovarian cancer survival were analyzed using Cox’s proportional hazards analysis, yielding an HR with a 95%CI; adjustments were made for age at diagnosis, histologic subtype/grade, and clinical stage with the addition of oral contraceptives and parity in density analyses. The proportional hazards assumption was confirmed using a log-minus-log plot. A *p*-value < 0.05 was considered statistically significant. Stata SEwas used for the statistical analyses (version 16.0. College Station, Texas: StataCorp.).

## Results

**Fig. 1:**
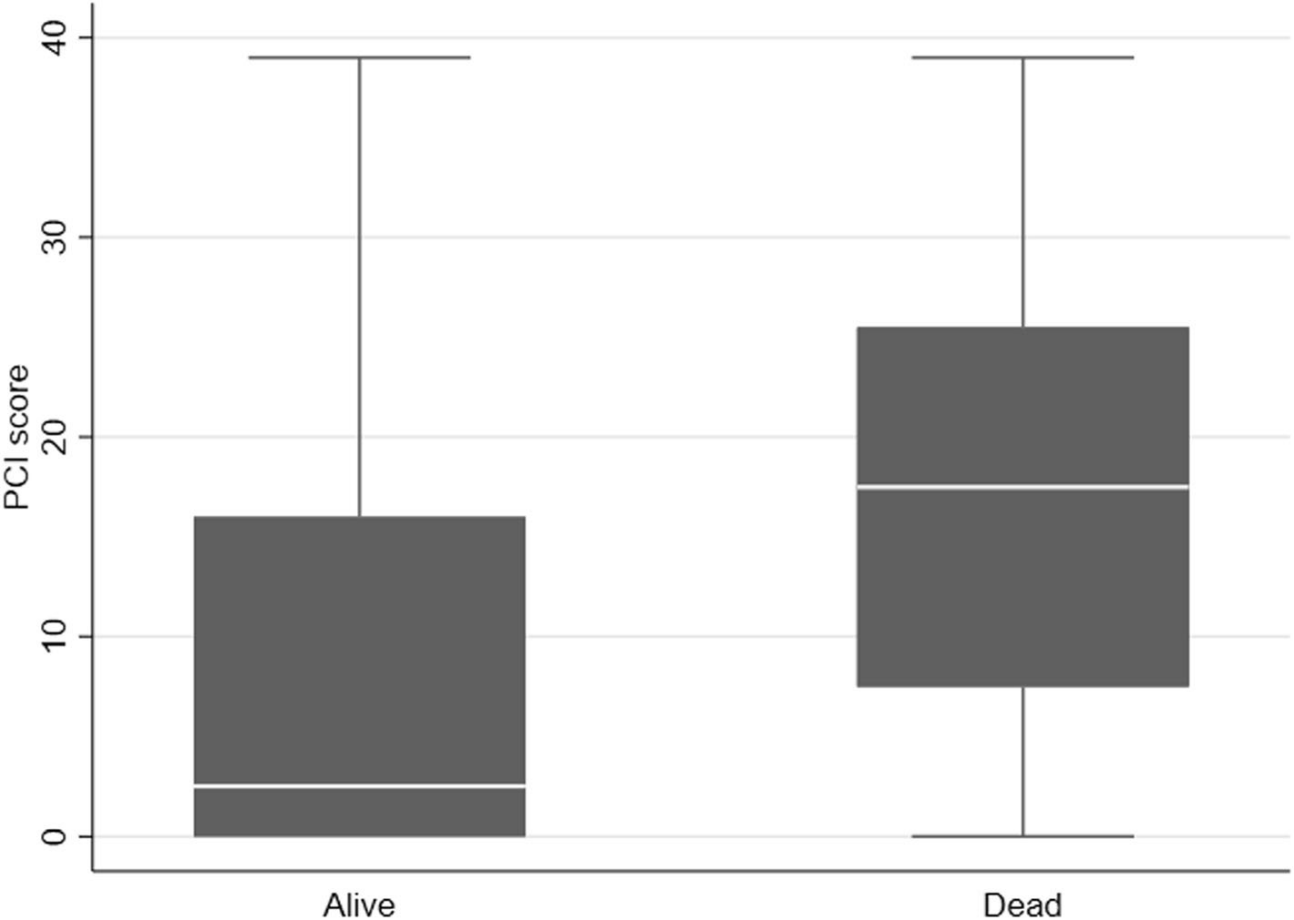
Distribution of PCI scores over ovarian cancer-specific survival

**Fig. 2:**
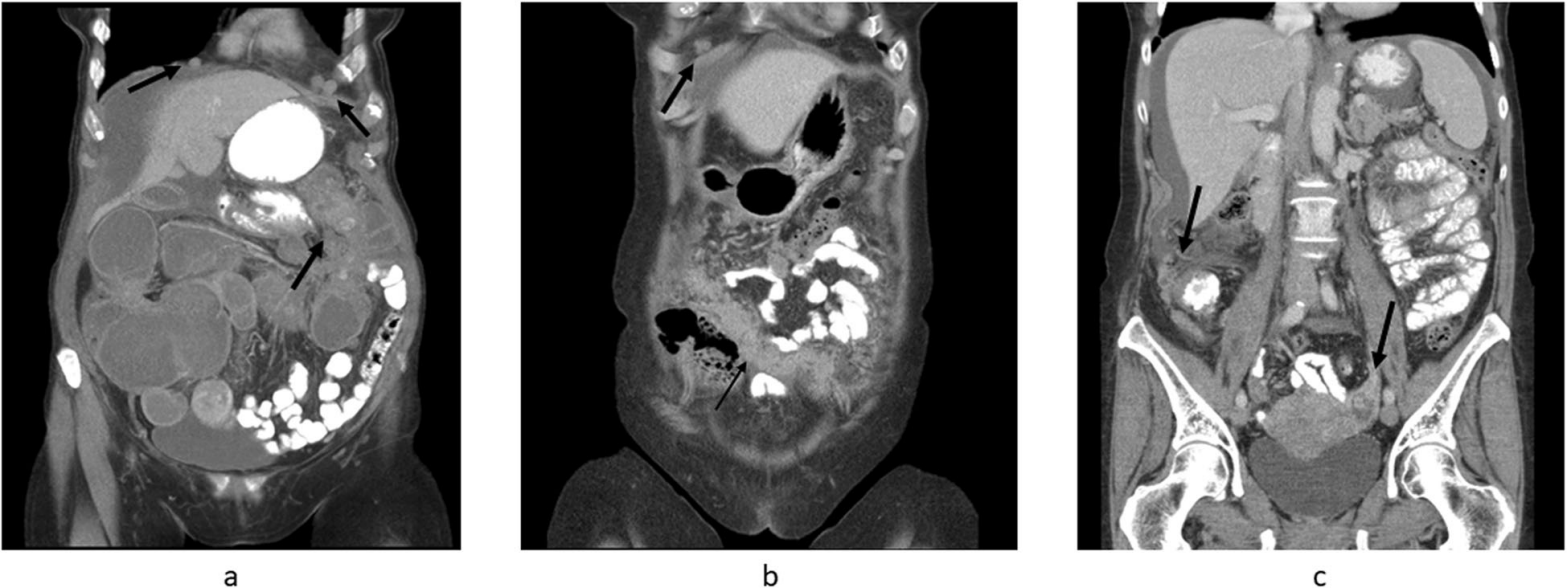
Peritoneal carcinomatosis and CPLN in women with ovarian cancer. **a** CT-image (coronary projection) showing bilateral enlarged CPLN and peritoneal carcinomatosis primarily affecting left side intestines, causing obstruction. **b** CT-image (coronary projection) showing ventral carcinomatosis, i.e. omental cake and enlarged CPLN. **c** CT-image (coronary projection, same examination as b) showing a left sided ovarian mass and diffusely spread carcinomatosis primarily affecting right sided colon

**Fig. 3:**
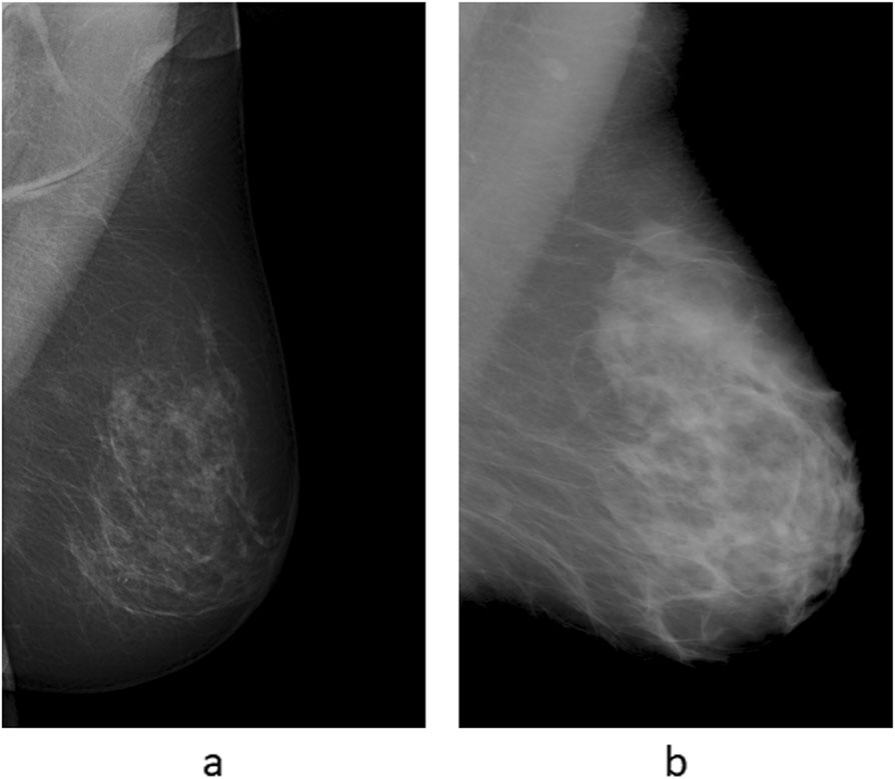
Breast density in women with ovarian cancer. **a** Mammography showing a fat involuted breast. **b** Mammography showing a dense breast

The imaging parameters and patient and tumor characteristics relating to ovarian cancer-specific survival are shown in Table 1. The mean difference between age at baseline and age at diagnosis was 11.3 years (range, 0.3–0.24). The distribution of PCI scores over ovarian cancer-specific survival is shown in Fig. 1. Radiological images illustrating peritoneal carcinomatosis, enlarged CPLN, and breast density is shown in Figs. 2 and 3.

### CT-peritoneal Carcinomatosis index

There was a statistically significant association between increasing CT-PCI and advanced clinical stage (OR_adj_ 1.26 (1.07–1.49), *p* = 0.007), adjusted for age at diagnosis and histologic type/grade (Table 2). There was no association between CT-PCI and histologic type/grade (OR_adj_ 1.02 (0.98–1.06), *p* = 0.434). Furthermore, higher CT-PCI was significantly associated with impaired OC specific survival (HR_adj_ 1.04 (1.01–1.07), *p* = 0.003), adjusted for age at diagnosis, histologic type/grade, and clinical stage.

**Table 2:** Peritoneal Carcinomatosis Index (PCI) in relation to ovarian cancer subtypes/survival

	Median (range)					
	Stage I	Advanced Stage	OR (95% CI)	*p*-val	OR_Adj_^a^(95% CI)	*p*-val
PCI	0 (8)	17 (39)	1.29 (1.10–1.53)	0.002	1.26 (1.07–1.49)	0.007
Observations			92		90	
	Low grade	High grade	OR (95% CI)	*p*-val	OR_Adj_^b^(95% CI)	*p*-val
PCI	2.5 (39)	14 (39)	1.02 (0.99–1.06)	0.170	1.02 (0.98–1.06)	0.434
Observations			92		90	
	Alive	Dead	HR (95% CI)	*p*-val	HR_Adj_^c^(95% CI)	*p*-val
PCI	2.5 (39)	17.5 (39)	1.05 (1.02–1.07)	< 0.001	1.04 (1.01–1.07)	0.003
Observations			94		90	

^a^Adjusted for age at diagnosis and histological type/grade

^b^Adjusted for age at diagnosis and stage

^c^Adjusted for age at diagnosis and stage and histological type/grade

### Cardiophrenic lymph nodes

**Fig. 4:**
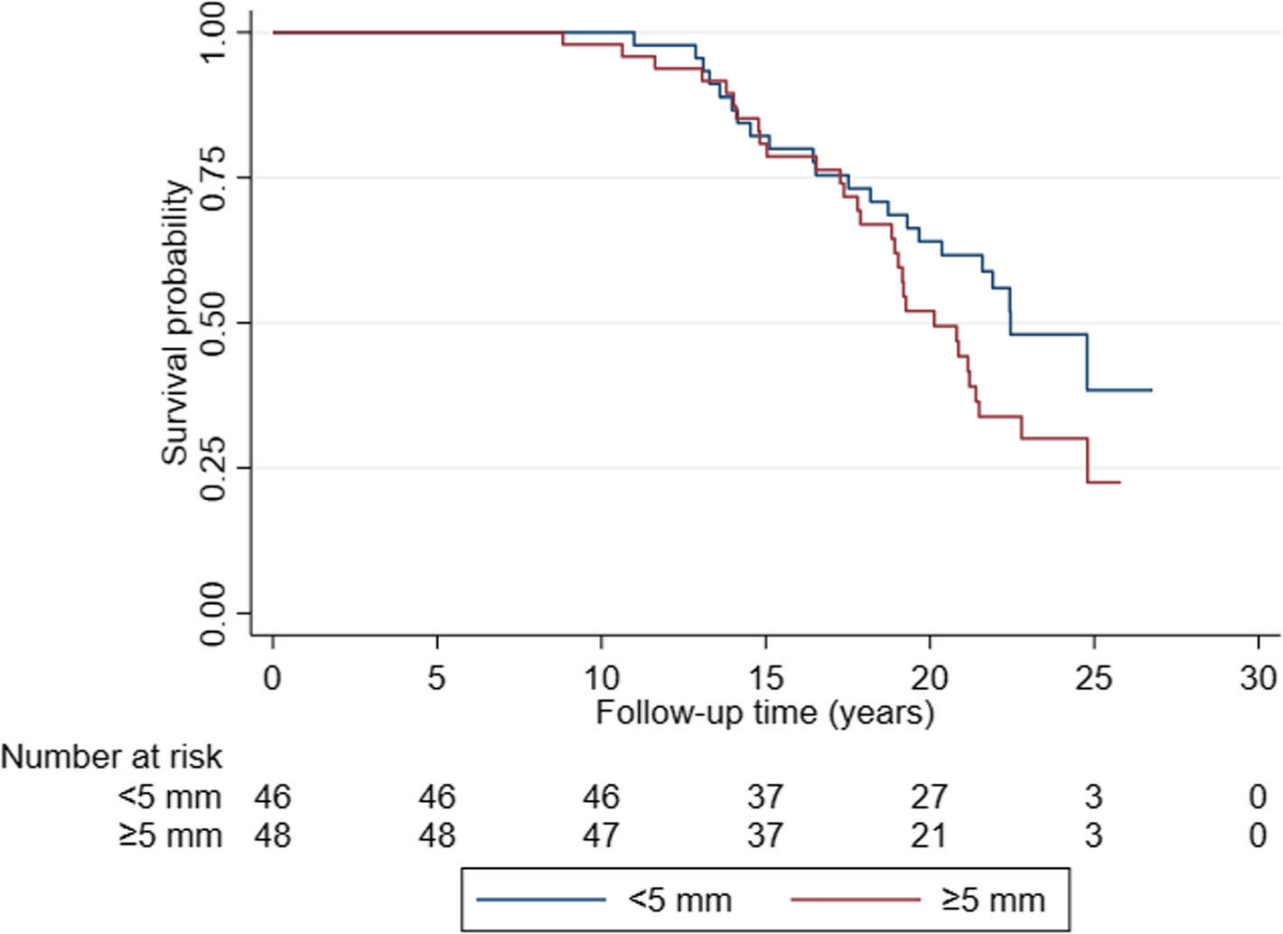
CPLN in relation to ovarian cancer specific survival

In crude analyses, there was a statistically significant association between enlarged CPLN and advanced clinical stage (OR 3.14 (1.10–10.57), *p* = 0.033) (Table 3). After adjustments, the association was in the same direction although not significant (OR_adj_ 2.13 (0.69–7.66), *p* = 0.173). There were no statistically significant associations between enlarged CPLN and histologic type/grade or OC specific survival. However, for both analyses enlarged CPLNs were more frequent with high grade tumors as compared to low grade tumors (57.1% vs. 41.7%) and more frequent with OC deaths vs. alive cases (57.7% vs. 42.9%). The non-significant difference in survival between the two groups of CPLN is further illustrated in Fig. 4.

**Table 3:** Cardiophrenic lymph nodes (CPLN) in relation to ovarian cancer subtypes/survival

	n (%)					
	Stage I	Advanced Stage	OR (95% CI)	*p*-val	OR_Adj_^a^(95% CI)	*p*-val
CPLN				0.033		0.173
< 5 mm	13 (72.2)	32 (43.2)	1.0 (Ref.)		1.0 (Ref.)	
≥5 mm	5 (27.8)	42 (56.8)	3.14 (1.10–10.57)		2.13 (0.69–7.66)	
Observations			92		90	
	Low grade	High grade	OR (95% CI)	*p*-val	OR_Adj_^b^(95% CI)	*p*-val
CPLN				0.149		0.181
< 5 mm	21 (58.3)	24 (42.9)	1.0 (Ref.)		1.0 (Ref.)	
≥5 mm	15 (41.7)	32 (57.1)	1.87 (0.80–4.36)		1.86 (0.75–4.62)	
Observations			92		90	
	Alive	Dead	HR (95% CI)	*p*-val	HR_Adj_^c^(95% CI)	*p*-val
CPLN				0.110		0.275
< 5 mm	24 (57.1)	22 (42.3)	1.0 (Ref.)		1.0 (Ref.)	
≥5 mm	18 (42.9)	30 (57.7)	1.57 (0.90–2.73)		1.40 (0.77–2.54)	
Observations			94		90	

^a^Adjusted for age at diagnosis and histological type/grade

^b^Adjusted for age at diagnosis and stage

^c^Adjusted for age at diagnosis and stage and histological type/grade

### Breast density

**Fig. 5:**
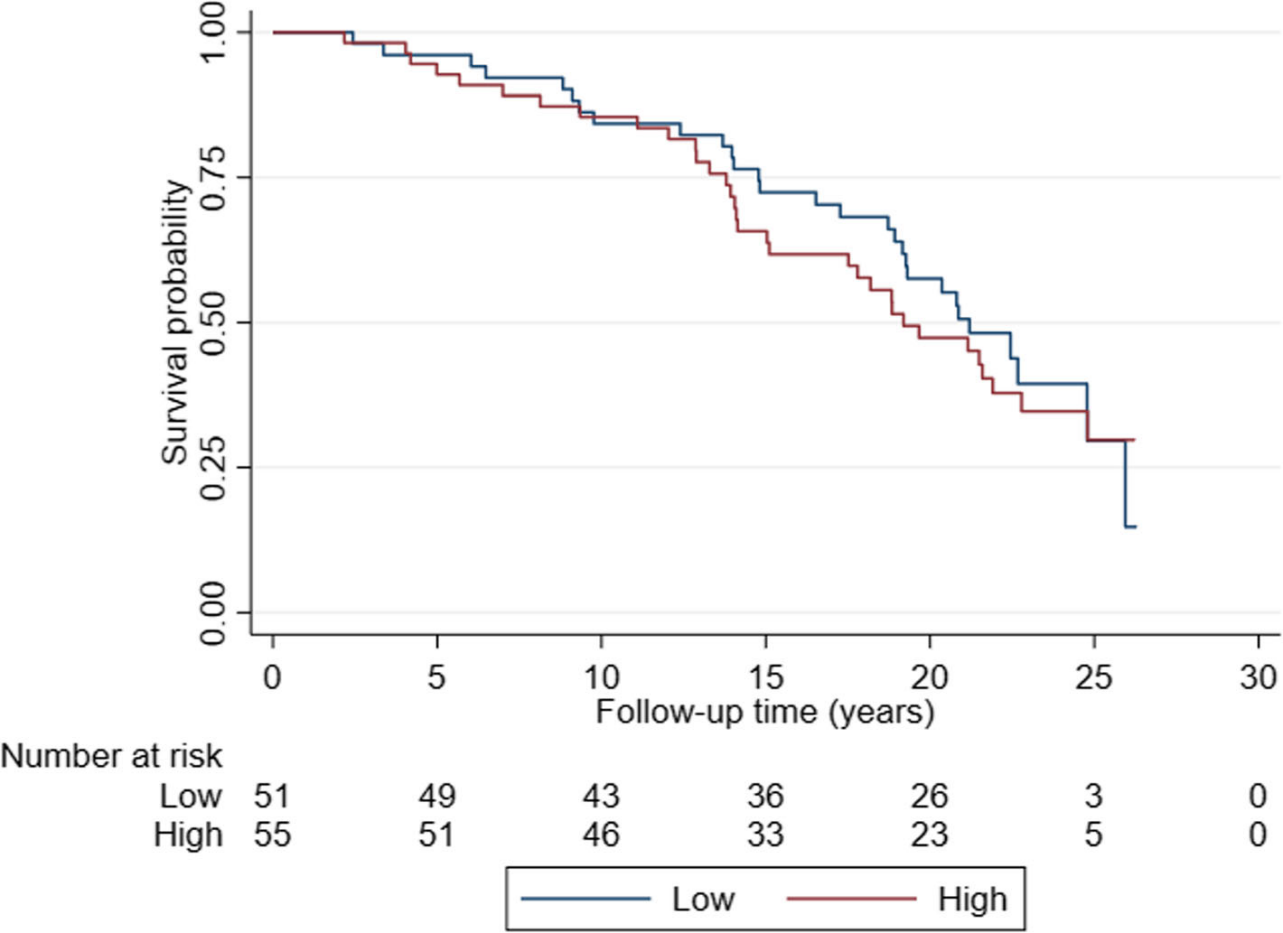
Breast density in relation to ovarian cancer specific survival

There were no significant associations between high BD and clinical stage (OR_adj_ 1.65 (0.57–4.78), *p* = 0.361) or histologic type/grade (OR_adj_ 1.13 (0.46–2.79), *p* = 0.787), adjusted for age at diagnosis, parity, and oral contraceptives. Also, high BD showed no relation to OC specific survival (OR_adj_ 0.90 (0.52–1.56), *p* = 0.708), adjusted for age at diagnosis, histological type/grade, clinical stage, parity, and oral contraceptives. The relation between the two groups of density and survival is further illustrated in Fig. 5. All analyses with adjustments are shown in Table 4.

**Table 4:** Breast Density (BD) in relation to ovarian cancer subtypes/survival

	n (%)					
	Stage I	Advanced Stage	OR (95% CI)	*p*-val	OR_Adj_^a^(95% CI)	*p*-val
BD				0.408		0.361
Fatty	11 (55.0)	38 (45.2)	1.0 (Ref.)		1.0 (Ref.)	
Dense	9 (45.0)	46 (54.8)	1.51 (0.57–4.02)		1.65 (0.57–4.78)	
Observations			104		102	
	Low grade	High grade	OR (95% CI)	*p*-val	OR_Adj_^a^(95% CI)	*p*-val
BD				0.950		0.787
Fatty	15 (48.4)	33 (45.8)	1.0 (Ref.)		1.0 (Ref.)	
Dense	16 (51.6)	39 (54.2)	0.97 (0.42–2.27)		1.13 (0.46–2.79)	
Observations			103		101	
	Alive	Dead	HR (95% CI)	*p*-val	HR_Adj_^b^(95% CI)	*p*-val
BD				0.659		0.708
Fatty	22 (50.0)	29 (46.8)	1.0 (Ref.)		1.0 (Ref.)	
Dense	22 (50.0)	33 (53.2)	1.11 (0.67–1.82)		0.90 (0.52–1.56)	
Observations			106		100	

^a^Adjusted for age at diagnosis, parity and use of oral contraceptives

^b^Adjusted for age at diagnosis, parity and use of oral contraceptives, stage and histological type/grade

## Discussion

In this study, we have identified significant relationships between CT-PCI, advanced clinical stage, and longtime follow-up ovarian cancer-specific survival. CPLN and mammographic density did not show a relationship with ovarian cancer subtypes or survival. From an imaging point of view, this demonstrates how structured reporting of peritoneal carcinomatosis can aid in clinical care.

Many factors affect the sensitivity of CT for detecting peritoneal carcinomatosis, such as lesion size, ascites, and technical parameters. However, CT is still considered the imaging method of choice for detecting ovarian tumors and peritoneal implants [20]. According to a previous meta-analysis on malignancies with peritoneal metastasis (of which 46% had a gynecological origin), CT is reported to underestimate carcinomatosis by 12–33% [21], but there was still a moderate to high agreement between CT-PCI scores and surgical-PCI scores (kappa = 0.49 to 0.96) [21]. Furthermore, we believe that patient outcome is particularly interesting to highlight and is likely more relevant than surgical findings as a standard of reference for imaging studies. Interestingly, CT-PCI can be used to preoperatively estimate the surgical challenges and postoperative complications in women with OC [8]. Also, Diaz et al. have shown that peritoneal disease quantified by CT-PCI in advanced-stage ovarian cancer patients correlated with presurgical CA-125 levels and 5-year survival [9]. This is in line with our results, showing increasing CT-PCI to be significantly related to impaired OC-survival, even after adjustment for histological subtype and clinical stage. Previous studies [8, 9] have addressed stage III and IV OC. However, we believe that it is important to include all OC stages since, in daily clinical practice, at the time of imaging, the clinical stage is unknown. Therefore, including all stages more adequately reflects the true clinical scenario. It would have been interesting to subdivide the CT-PCI to further investigate if certain strategic abdominal areas were over- or under-represented. However, this was not possible due to the small sample size.

We could not establish an association between CT-PCI and histological subtypes, which is in line with the findings of a previous study (although comparing serous and non-serous tumors) [9]. However, there was a significant relationship between increasing CT-PCI and advanced stage, which, to the best of our knowledge has not been previously studied and is coherent with our results on increasing CT-PCI and impaired survival. Hence, the PCI-score could be useful to consider during several steps in clinical management.

A drawback of this study is the lack of information on potential neoadjuvant therapy, surgical method, and surgical outcome, especially since adequate surgical cytoreduction is the most important independent factor affecting survival in epithelial ovarian cancer [22, 23]. However, the methods of surgery and the reporting of surgical outcomes vary over time in the MDCS, which would make this information difficult to interpret and classify. Furthermore, the survival analyses were adjusted for histological subtype/grade and clinical stage, both strongly contributing factors in selecting the method of surgery and treatment and could be proxy variables for surgical method and outcome. Lastly, the important aspect here is not the well-known fact that increased carcinomatosis is linked to surgical difficulties and more severe stages and outcome, but rather the important fact that an image score has the potential to reliably quantify carcinomatosis with a clear link to outcome, independent of time period.

At our institution, a prospective trial on women with ovarian cancer is planned, including scoring of CT-PCI, CPLN (by at least two radiologists) and surgical-PCI, registration of patient and pathology factors at the time of diagnosis, medical and surgical treatment, and overall outcome. Taken together, evaluated and quantified CPLN and carcinomatosis through imaging can help clinicians in the surgical and prognostic dilemma early in the diagnostic procedure.

CPLN is an interesting imaging parameter since it can be retrieved from the very same CT as the PCI-score. In this study, no significant associations between CPLN size and the clinical parameters were found in the adjusted analyses. Interestingly though, for all analyses enlarged CPLN signified a more aggressive disease (advanced stage, high grade tumors, and OC death), which is consistent with previous knowledge [10]. Other malignancy criteria for lymph nodes (border contour and signal intensity characteristics) than the somewhat blunt size criteria have proven predictive of lymph node status in rectal cancer [24]. Additional studies with larger sample size and with additional malignancy criteria (beyond size) may be of value to further establish these associations.

Regarding BD, Wernli et al. have recently reported an association between increased BD and a moderately increased risk of ovarian cancer in women of 50–59 years of age [14]. To our knowledge, there is no previous study on BD and OC survival. Our hypothesis was that high BD would be associated with more aggressive OC subtypes and impaired survival; however, this could not be established. One potential reason for this is that the time between screening mammography and OC diagnosis varied up to 2.5 years, hence the mammographic imaging parameter and the OC diagnosis are not from the same time point, and the BD might have been altered during this period. Unfortunately, BRCA status was not available in the MDCS cohort, which would have been valuable considering the link to both ovarian and breast cancer [25].

This retrospective cohort study is limited by the small sample size, mostly because only digital images could be used, and results should be interpreted with caution. However, there was no large difference in the distribution of clinical stage between the group with missing data as compared to the group without missing data (regarding PCI/CPLN and density groups), hence we believe the risk of selective bias to be very low. In addition, the images were performed on different radiology systems with varying image quality due to the long-time period. On the other hand, the long observation time is also an advantage considering follow-up time for survival information. Each of the three imaging parameters (CT-PCI, mammographic density, and CPLN) was estimated by a single radiologist, which does not allow for an analysis of inter-reader variability and reproducibility. However, the purpose of this study was no to create cut-offs for survival probability, but rather to study the direction of influences of certain imaging parameters that could be of value for future study and potential implementation in structured CT reports. As highlighted by Laghi et al. [21], the CT-PCI is a challenging interpretation for radiologists since the score has some uncertainties (e.g., when it comes to abdominal area and lesion characterization), which might be difficult to score correctly. As a future prospect, it would be valuable to develop a more straightforward score, but this was beyond the scope of this study. However, given the uncertainties of the score, it is even more interesting that we were able to show a clear association with OC survival in this study.

Our results have several clinical implications. One of the very first steps, when a woman is presenting with a suspicion of ovarian cancer, is to perform a CT scan. Therefore, the structured information gathered at imaging might impact early clinical management, such as a high CT-PCI score prompting the clinical management at a specialist clinic for ovarian cancer, or to be prepared for an advanced stage disease with both surgical and prognostic challenges. Our results fit well with the structured surgical report from the European Society of Gynecological Oncology (ESGO), where surgical PCI is included in the surgical report, and as a future goal, the CT-PCI may be added to this chart.

## Conclusions

We have identified significant relationships between increasing CT-PCI and important clinical parameters, such as advanced stage and impaired OC specific survival. When taken together, imaging parameters have the possibility to aid in the clinical care of women with ovarian cancer.

## Data Availability

The datasets used and/or analysed during the current study are available from the corresponding author on reasonable request.

## Abbreviations

BD: Breast density
BI-RADS: Breast imaging report and data system
CI: Confidence interval
CPLN: Cardio phrenic lymph nodes
CT: Computer tomography
OC: Ovarian cancer
PC: Peritoneal carcinomatosis
PCI: Peritoneal carcinomatosis index
